# Deep learning for EEG-based prognostication after cardiac arrest: from current research to future clinical applications

**DOI:** 10.3389/fneur.2023.1183810

**Published:** 2023-07-24

**Authors:** Frederic Zubler, Athina Tzovara

**Affiliations:** ^1^Department of Neurology, Spitalzentrum Biel, University of Bern, Biel/Bienne, Switzerland; ^2^Institute of Computer Science, University of Bern, Bern, Switzerland; ^3^Department of Neurology, Zentrum für Experimentelle Neurologie and Sleep Wake Epilepsy Center—Neurotec, Inselspital University Hospital Bern, Bern, Switzerland

**Keywords:** deep learning, EEG, prognostication, coma, cardiac arrest

## Abstract

Outcome prognostication in comatose patients after cardiac arrest (CA) remains to date a challenge. The major determinant of clinical outcome is the post-hypoxic/ischemic encephalopathy. Electroencephalography (EEG) is routinely used to assess neural functions in comatose patients. Currently, EEG-based outcome prognosis relies on visual evaluation by medical experts, which is time consuming, prone to subjectivity, and oblivious to complex patterns. The field of deep learning has given rise to powerful algorithms for detecting patterns in large amounts of data. Analyzing EEG signals of coma patients with deep neural networks with the goal of assisting in outcome prognosis is therefore a natural application of these algorithms. Here, we provide the first narrative literature review on the use of deep learning for prognostication after CA. Existing studies show overall high performance in predicting outcome, relying either on spontaneous or on auditory evoked EEG signals. Moreover, the literature is concerned with algorithmic interpretability, and has shown that largely, deep neural networks base their decisions on clinically or neurophysiologically meaningful features. We conclude this review by discussing considerations that the fields of artificial intelligence and neurology will need to jointly address in the future, in order for deep learning algorithms to break the publication barrier, and to be integrated in clinical practice.

## 1. Introduction

Cardiac arrest (CA) is one of the leading causes of coma worldwide, with around 19 million cases per year. The majority of patients who survive a cardiac arrest are initially comatose, as a result of global ischemia. Patients who would have died from a cardiac arrest are nowadays receiving advanced treatments and are more likely to survive (1, 2). As the brain is more susceptible to ischemia than other organs (2–4) the most determinant outcome of coma after cardiac arrest is the ischemic hypoxic encephalopathy (HIE). In the recent years, it has become increasingly important to have early and accurate predictions of patients' outcome to avoid futile treatment, better allocate resources and to inform patients' families. Currently, outcome prediction relies on a multi-modal approach comprising clinical and paraclinical tests (4). Because it directly assesses the neural activity of the brain, electroencephalography (EEG) is one of the most widely used and accurate methods for prognostication in HIE after CA (5, 6).

Currently, EEG is analyzed visually by a Neurologist or Neurophysiologist. This procedure requires a specific expertise, is time consuming, and is prone to subjective assessments (7, 8). Moreover, even though several EEG patterns are associated with favorable or unfavorable outcome, there is a relatively large amount of patients that remain in a “gray-zone”. These are patients for whom EEG is not able to make an accurate prognostication, or might be discordant to other modalities. This case may occur for example when the EEG is indicative of favorable outcome according to current visual criteria (e.g., a continuous reactive background without periodic pattern) but the patient still fails to awaken after several days, and/or other modalities such as neuroimaging or biological markers (e.g., neuron-specific-enolase) are suggestive of poor outcome.

Finally, visual EEG analysis is by definition limited by the capability of the Neurophysiologist for interpreting a multidimensional time series (9). Potential crucial features of the signal, for instance statistical properties or fine-grained patterns of activity might be oblivious to the human eye, and therefore do not contribute to the prognostication. There is a clear need for novel diagnostic and prognostic methods. Computational techniques aim at analyzing EEG signals in a fast, automated, and objective way, possibly also increasing the yield of EEG by detecting new clinically relevant features. Due to its tremendous success in various fields such as visual object recognition or automatic translation, deep learning is a natural candidate computer-based EEG analysis. While deep learning has been applied to EEG in various clinical and non-clinical settings (10, 11), in this review, presented in a narrative review format, we will focus on its use for prognostication after CA.

## 2. What is deep learning and why use it for EEG analysis

### 2.1. General principles of classification

**Figure 1:**
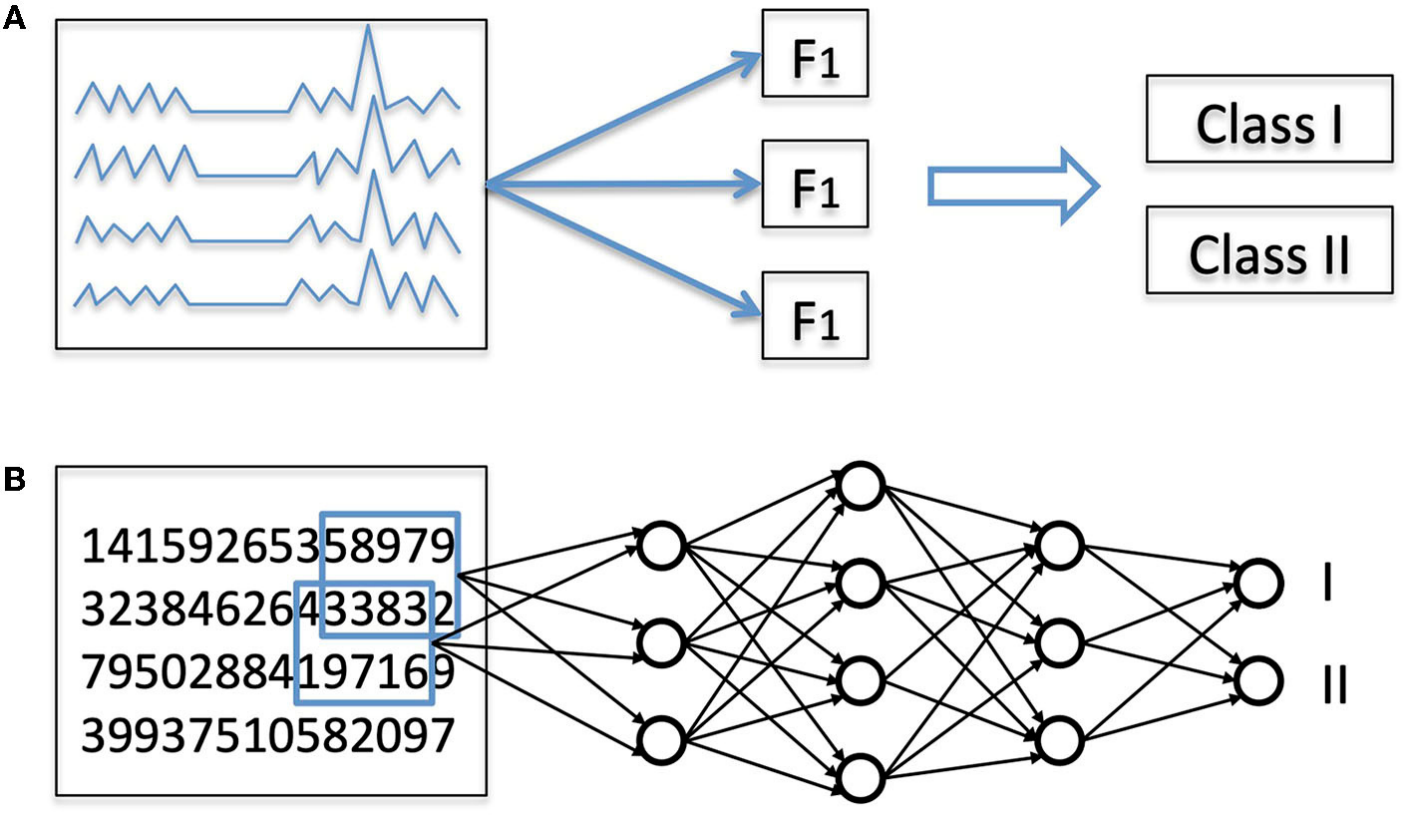
**(A)** General principles of classification of EEG signals. Almost every classification task is a two-step process: first, extraction of features, and second, actual classification based on these features. **(B)** Classification with a deep learning (DL) network. In a DL network, both steps are performed by the same hierarchical network, which takes as input a numerical representation of the data and outputs the probability associated with each class.

To understand the potential advantages of deep learning compared to other computer-based approaches, it is useful to think about what it means to classify an input, whether an image, a sound, or a biological time series such as an EEG. First, one has to recognize relevant features, namely properties of the data that will help perform the classification (Figure 1A). If confronted with a photo of an animal, one might recognize a large animal with four legs, with stripes, or with a long neck. Then, based on our previous zoological knowledge, we use this information to classify the animal, that is, to recognize it as belonging to the class of zebras or the class of giraffes. Errors can occur at several points along this process: the quality of the photo might be too low, one might falsely interpret stripes as shadow from branches, or correctly recognize stripes on the animal but ignore the existence of zebras. Of note, sometimes the absence of a certain feature is important for classification (e.g., the absence of stripes can help in identifying a horse in a group of zebras).

The same principles apply to the visual analysis of EEG in comatose patients. Using for instance the item listed in the American Clinical Neurophysiology Society (ACNS) terminology (12), one can describe the background continuity, frequency, amplitude, or the presence of periodic or rhythmic elements or epileptic seizures. Then, based on the list of identified elements, one applies a classification system. For instance, it has been shown (5) that a continuous (feature 1) and reactive (feature 2) EEG background with posterior to anterior gradient (feature 3) and in absence of periodic rhythmic element (absence of feature 4) was associated with a favorable outcome. Other classification systems can be applied to the same set of features. For instance, tolerating the absence of feature 1 or 3 increases the sensitivity for favorable outcome (13). As was the case with animal pictures, errors can apply at different points also in the case of EEG evaluations. For example, a technical issue during an EEG recording might artificially reduce its amplitude; EEG elements can be misidentified (for instance electrocardiogram artifacts mimicking periodic discharges); or a clinician might ignore that sedation can alter the continuity of EEG traces.

Computational analysis methods have been used in the field of EEG to help either with feature recognition, classification, or both. For instance, simple signal processing techniques can automate frequency analysis, or, in other words, the decomposition of an EEG signal into components with different frequencies. The relative importance of the different frequency components at different time steps can be displayed in a condensed matter called a spectrogram. This type of representation is very useful for long-term monitoring, so that a human observer can rapidly identify time points where an EEG signal might change, for instance due to awakening, sedation, or epileptiform activity (14). However, the interpretation of these features (the “real” classification), lies by the human expert. The advantage of computing and displaying features for a long EEG recording is to save time, which might be of the essence to start a treatment, but does not necessarily increase the yield of EEG (15, 16).

Alternatively, feature recognition can be performed by humans while classification is done by a computational algorithm. Features described in the ACNS terminology are numerous, and their possible combinations even more so (12). In a prognostication study on critically-ill patients with impaired consciousness, the presence of different features was scored visually by EEG experts. In a second step, classification was performed based on these features with a random forest algorithm, a machine learning technique whereby 500 decision trees were generated based on the data and voted to predict the clinical outcome (17).

Last, both approaches can also be combined. EEG quantitative features (mainly based on frequency analysis) and random forest were used to predict outcome after cardiac arrest (18) or traumatic brain injury (19). The same group also hand crafted quantitative features mimicking the ones recognized by humans (such as continuity, irregularity etc.) which were automatically used for classification, as an attempt to emulate a neurologist or electrophysiologist (20). By contrast, other groups deliberately used features properties of the EEG signal that are not easily quantifiable without computational techniques such as synchronization measures applied on resting state (21) or auditory evoked EEG signals (22).

In all the studies mentioned above, EEG features were specified ahead of time by humans: even when computational techniques were used to analyze the EEG signals, the nature of the features that were computed (e.g., which frequency bands, or which synchronization measures) was specified a priori by a human expert. This approach is commonly referred to as “feature engineering”. The advantage of this approach is that we can benefit from 50 years of experience gathered by electroencephalographers. The limitation, however, is that explicitly crafting the features is by definition limited by prior knowledge. There is another possible approach, where features are defined by an algorithm. This approach is called “feature learning”. Its main advantages are that it is data-driven and not limited by a priori assumptions. In deep learning, the same algorithm performs simultaneously feature extraction and classification (Figure 1B).

### 2.2. How deep learning works

**Figure 2:**
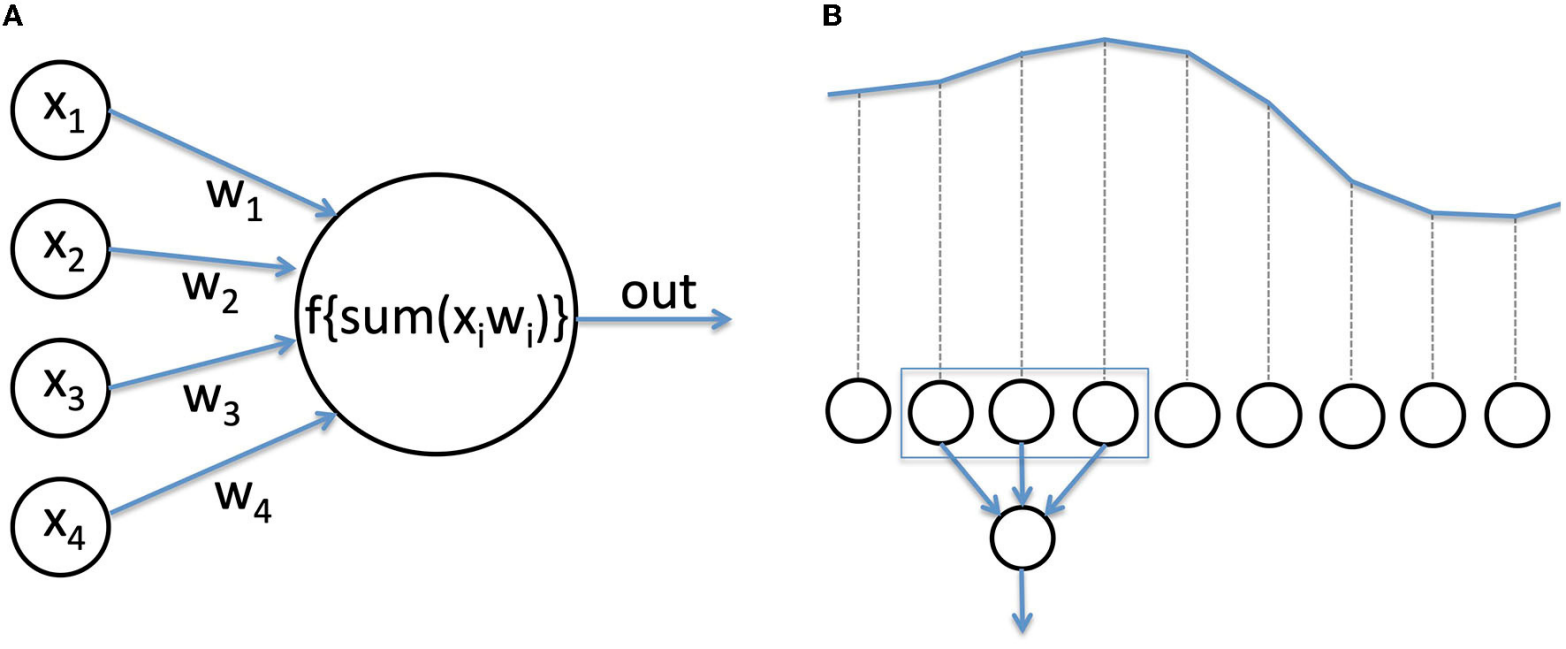
**(A)** An artificial neuron. Neurons are the computational units of a deep neural network. Inputs are multiplied by a weight, added, and passed through a function to produce the output. **(B)** For EEG analysis, the voltage recorded at each time step serves as numerical input for the network.

Deep learning (DL) is a class of hierarchical algorithms (the “networks”, or “models”) composed of multiple processing units (“neurons”) (23). Each neuron receives several numerical inputs, which are multiplied by weights, and summed up. This sum is then passed to a function to produce the output (Figure 2A). Usually, neurons of the last layer of a network serve as general output for the network, or a “prediction”. The name neuron comes from the resemblance with biological neurons, in which inputs are provided by other neurons or receptors, while the weights would be synapses with variable strengths, the weighted sum of inputs approximates the membrane potential, and the non-linear function represents the generation of action potentials. Using artificial neurons to perform a labeling task is not a new idea (24). Their recent success in practice is due to novel methods of training as well as hardware progresses, allowing training of networks with a large number of layers (hence the term “deep”) (25). Currently, deep learning is state of the art in domains such as image processing, or automatic translation, and has often become a synonym for Artificial Intelligence (AI).

For image processing, the input to a neural network is the value of the red, green, and blue component of each pixel. In the case of EEG, the input is usually a time series of voltage values recorded over time (Figure 2B). To become efficient in analyzing new input data, a network has to be trained, that is, the weights of the neurons need to be adjusted (hence the term “learning”). A detailed tutorial on training methods lies beyond the scope of this review. In a nutshell, during training for a classification task, the network is repetitively presented with input data, which are passed through all layers, and the final output of the network is compared with the real class (e.g., class I = patient had a favorable outcome, class II = patient had an unfavorable outcome). If the prediction is correct, the weights of the network are increased (the prediction is reinforced), and if wrong, the weights are decreased.

A convolutional neural network (CNN) is a specific type of DL architecture in which neurons in the first layer are not simultaneously connected to all of the inputs, but are swept through the input, acting as a kind of filter (i.e., via a convolution) (25). This procedure saves computational time for large data and allows for useful properties such as position invariance. When the complexity of an artificial neural network is sufficiently high, the network can approximate any function; CNNs can therefore perform both feature extraction (i.e., approximate those functions that would compute relevant features of the input data), and at the same time, classification.

## 3. Review of the literature

### 3.1. Methodology

A search was performed in PubMed using the following terms: *(deep learning OR artificial neuron OR neural network OR convolutional OR CNN OR DNN OR RNN OR LSTM) AND (Coma OR cardiac arrest OR ICU OR HIE OR hypoxic OR anoxic) AND (EEG OR electroencephalogram OR electroencephalography)*. 114 articles were found, of which six were original studies at least partially devoted to the use of deep learning for prognostication after cardiac arrest in adult humans based on EEG recordings. Using references, another relevant peer-reviewed study was found, resulting in the inclusion of seven studies for the present review. We did not perform searches using other engines, nor did we include papers published only in a repository or as a PhD thesis.

The identified studies were evaluated in terms of the network architecture that they used, the total number of patients, the main task that deep learning aimed at solving, the classification performance, and whether any feature visualization techniques were used.

### 3.2. Summary of the literature search

Five of the existing studies on the use of deep learning for coma outcome prognosis used a simple convolutional neural network (CNN), albeit with very different architectures:

Van Putten et al. (26) used a network with one convolutional layer to analyze a monocentric data set at various time points after cardiac arrest (CA); the training set contained 230 patients, while the test set of 50 patients. The area under the ROC curve (AUC) of the model was 0.89 at 12 h after CA and 0.76 at 24 h.

Tjepkema-Cloostermans et al. (27) used a Visual Geometry Group (VGG) architecture (3). This consists of a deep CNN with 13 convolutional layers, with a progressively decreasing size of convolutions. Training and internal validation was performed on 661 patients from two hospitals, external validation on 234 patients of three other hospitals. The performance for both validation sets at two different time points was between 0.86 and 0.92. Simultaneously incorporating data from two different time points did not significantly modify the prediction performance.

The same group (18) analyzed a largely overlapping data set using the same convolutional network in the context of a comparison study between different methods (see below). The AUC of the network was between 0.88 and 0.90.

Jonas et al. (28) used a reduced version of the VGG network (“t-VGG”) with six convolutional layers to analyze a monocentric data set (training set 213 patients, test set 53 patients). An overlap of 75% between epochs was used to increase the training samples. The mean EEGs latency was 20 h after CA. The performance was 0.89 for version with additional all-to-all layer, and 0.9 without (t-VGG-GAP).

The t-VGG-GAP architecture was then used for outcome prediction in multicentric cohort of patients with different etiologies of coma (of which 30% after CA) (29). The authors used five-fold cross validation (286 patients for training and 72 for testing). The performance was 0.72 for predicting survival and 0.70 for predicting a favorable outcome (defined as survival without major disability). Of note, the same architecture could be trained for predicting the cause of coma.

Two other studies were found that used a more complex architecture and/or paradigm:

Zengh et al. (30) used a CNN for feature extraction in single epochs, which was then fed to other DL networks (“long short time memory”) to introduce time dependency between epochs, and was then combined with clinical data for final prognostication. 1,038 patients from 7 hospitals were analyzed (five-fold cross correlation). The AUC was 0.83 at 12 h after CA and 0.91 after 66 h.

Aellen et al. (22) focused on short EEG epochs (550 ms) around auditory stimuli, in contrast to the other studies where resting state EEG was used. The input data used in this study to train the neural networks are slightly different from what was used in the previous studies (following auditory stimulation vs. resting state). Nevertheless, the end goal is within the same scope, in predicting outcome from post-anoxic coma via EEG signals. The data set consisted of 136 patients from four hospitals. The AUC was 0.70, whereas the positive predictive value for favorable outcome was especially high (92%). The discriminative capability of the classifier remained at similar levels on a subgroup of gray zone patients with uncertain outcome prognosis based on existing clinical criteria.

## 4. Discussion

Despite the use of different architectures, the performance of previous work assessing coma outcome based on CNNs was remarkably high and very similar. This point was explicitly investigated in Jonas et al. (28): optimizing the hyperparameters (size of EEG epoch, size and number of filters, number of convolutional layers) or using other architectures from the EEG literature only modified the network's performance by a few percent points. This finding is reassuring for future use of CNNs in the medical practice, as the same architecture can probably be used in many different clinical tasks.

Overall, the performance for prognostication after CA was high, with AUC around 90% in the different studies. When the networks were directly compared to other computational methods, their performance was equivalent, and the DL algorithms were more resistant to noise (18). When applied to a group of patients with various etiologies of coma (29), the network's performance dropped (AUC 70%), whereas visual scoring of specific features together with a random forest classifier achieved an AUC of 80% on the same data set (17). Note however that the combined visual feature/random forest approach leveraged the knowledge of EEG experts with specific qualification in ICU EEG, and was allowed to use information concerning EEG reactivity, namely the modification of the EEG background after auditory of somatosensory stimulus, whereas the DL algorithm was trained exclusively on resting state EEG (in absence of stimulation). By focusing on EEG during auditory stimulation, DL could find new predictors of favorable outcome also for patients in the gray-zone (patients who cannot be classified with current markers) (22).

The use of deep neural networks for coma outcome prognosis is appealing for several reasons. First, they are highly performant; second, unlike the majority of existing clinical tests, they can be informative of both positive and negative outcome; third, they do not suffer from subjective evaluations of EEG data; fourth, when coupled with auditory stimulation, they can additionally provide predictions of coma outcome for patients that are in a clinical “gray-zone”, with indeterminate prognosis; fifth, with sufficient training data, a DL-based algorithm could theoretically be expanded to recognize other important clinical aspects that merely predict outcome without the need to handcraft new features.

### 4.1. Bias and interpretability in deep learning for EEG: a double-edged sword

Despite its advantages, several challenges need to be addressed before DL can be deployed and used in clinical settings. First, as DL networks learn based on data, they can reproduce existing biases in the data at scale (31). The medical field is full of examples of bias, starting already from data collection. Scalp EEG measurements may be biased against several racial groups, because the measurement itself may be inaccurate (32). For instance, EEG devices may not be able to accommodate coarse and curly hair, resulting in poor data quality (32, 33). Although this limitation exists also in the case of visual EEG evaluations, it may be particularly concerning when EEG signals are analyzed with the use of AI black-box like approaches, where decisions reached by a network may be due to poor data quality instead of intrinsic and desired features of the EEG response. This is especially problematic if members of underrepresented patients cohorts are further excluded due to selection bias, which has been previously described following a lack of written informed consent, requested by ethical committees (34).

After data collection, certain patient profiles may exhibit particular characteristics in their EEG signals, for example due to age, temperature treatment, concomitant pathologies such as epileptic seizures, infection, metabolic disturbances, or because of the use of anesthetics, which drastically alter patterns of spontaneous EEG activity. Some computational approaches for coma outcome prognostication performed poorly when applied to patients with epileptiform activity (35, 36). Moreover, in one of the DL studies mentioned above, epileptic seizure but also metabolic encephalopathy in case of liver insufficiency introduced errors in the classification (28). In the same study it was shown that introducing EEGs recorded during physiological sleep in the training set changed the specificity and sensitivity for detecting poor outcome in coma. Also, training the same architecture with patients with other etiologies of coma allowed for specific patterns not typically seen in patients with severe HIE (frontal generalized rhythmic delta activity) to be recognized as relatively benign.

One of the main limitations of current studies using DL for clinical EEG analysis is the focus on a single problem [e.g., prognostication, detection of epileptiform activity (37), scoring of sleep stages (38) or artifact detections (39)] and the use of highly specific training datasets, often with manual selection of artifact-free epochs. This approach may artificially increase the DL performance, which may be appealing to reviewers of scientific articles, but often at the cost of generalization in a real world settings.

Before being deployed in a clinical environment, any AI algorithms should therefore be trained on enough data so that all potential cases are well represented. In the country of the authors of this review (Switzerland), data sharing is actively promoted by funding agencies, but, in practice, it can be difficult to openly share clinical data due to local regulations. Algorithms should also be extensively tested in sub-groups of coma patients, to ensure that they perform as expected not only at group level, but also within individual sub-populations. This implies, among others, the tedious work of inspecting false positive and false negative rates, to understand the reason for the misclassification (29).

Although the question of bias should not be taken lightly, the use of AI technologies for coma outcome prognosis can in fact help in overcoming human biases. These may arise at several levels, the most evident of which concerns the visual analysis of EEG signals which is inherently prone to subjective judgement. Although interrater agreement is generally high for very malignant EEG patterns, it is only moderate for malignant patterns, and quite low for assessing EEG reactivity, which is a predictor of good outcome (7, 40). In particular, interrater agreement can be quite low between junior and senior neurophysiologists (8) or between neurophysiologists and EEG technologists (40). AI-based assessments of EEG signals can assist in overcoming these cognitive biases, by providing reproducible and objective assessments of EEG signals. They can also assist in homogenizing EEG evaluations across hospital centers, and levels of training in medical personnel.

Although AI has the potential to reduce cognitive biases resulting from subjective judgement, human biases may persist and be even amplified with the use of AI. These may be particularly present when interpreting and integrating results generated by AI algorithms. As new algorithms will necessarily learn from past data, they may be prone to propagating self-fulfilling prophecies, regarding the decision to withdraw life-support. Moreover, they may carry human biases that are related to clinical decision-making and patient assessment (31). Future initiatives will need to develop best practices that can be implemented to mitigate risks of biases and ensure transparency in novel AI applications.

Another important requirement is explainability. In the field of prognostication, and more generally in healthcare it is of crucial importance that humans can understand the rationale for a decision reached by an algorithm (41). This is crucial, first, in order to explain a medical decision to the patient and their family, and second, to help detect and mitigate some of the biases mentioned in the previous paragraphs. It is reassuring that most studies on DL-based prognostication include techniques to improve interpretability, or other control analyses. For instance (28, 29), used a so-called gradient method, which highlighted which part of the EEG signal was discriminative for each class. Interestingly, some patterns which were relevant for the network were similar to the ones used by human experts such as a suppressed background or spiky transients for unfavorable outcome; and continuous alpha or theta activity with posterior-anterior gradient, or generalized rhythmic delta activity for favorable outcome. With this type of methods, clinicians could evaluate on a patient-by-patient basis, whether a given prognosis was based on meaningful EEG features or whether it was instead driven by random interpretation of non specific parts of the EEG signal, or even artifacts. In an alternative approach (27), displayed the distribution of the network's output for sub-categories of EEG based on visual patterns. This analysis also showed good concordance with visual interpretation of EEG signals. Last, Aellen et al. (22) assessed correlations between the outcome prediction of the neural network for each patient and features of neural synchrony or complexity, which have been previously found to indicate chances of coma outcome, and of conscious processing (42).

### 4.2. Future directions: incorporating AI solutions into the clinical practice

Despite their strong potential, AI-based solutions for coma outcome prognosis are not yet integrated into the clinical practice. Among the challenges that will have to be resolved lay first of all questions of reproducibility. Before deployment, AI-based techniques will need to be systematically assessed across several hospitals, to ensure that results generalize and are robust beyond variations in local clinical practices, treatments, or medical decision-making. Importantly, as the guidelines for treating post-anoxic coma patients are constantly being updated, it would be important to demonstrate that any AI algorithms perform well despite local variations, for example due to anesthetics, blood pressure or targeted temperature management treatment (43–45). As training of AI algorithms requires extensive amounts of data, which are not always possible to share across hospitals, future applications could also examine the use of federated learning frameworks, which train AI algorithms locally, using consensus models, without the need to exchange data (46).

Another major challenge is that of digital literacy. AI algorithms for neuro-critical care need by definition to encompass two different fields: that of machine learning and neurology. To ensure a practical implementation, several challenges will need to be addressed. AI algorithms will need to be developed and deployed as a “plug-and-play” solution, that can be easily used by non-experts in AI or computer programming. At the moment, applying AI algorithms on EEG data requires advanced programming, machine learning, and data management skills, and also complex Graphics Processing Unit (GPU) setups. All these factors impede the routine use of AI by medical staff.

On the other side, the field of machine learning also needs to approach the clinical practice, by encouraging the development of AI techniques suitable for physiological signals. This implies a change of mindset. First, novel AI algorithms are needed for EEG signals that are as powerful as those developed for images or text, but taking into account the physiology and particular characteristics of EEG. Progress in the field of deep learning for EEG has given rise, in the recent years, to strong networks that include time-domain convolutions, and that have relatively low numbers of parameters and can learn from limited amounts of EEG data (47). Second, the nature of EEG signals needs to be considered when assessing the performance of AI algorithms. The field of machine learning oftentimes over-emphasizes network performance, and focuses on improvements that boost accuracy, which lead to impressive performance metrics for images, videos, or text. However, when dealing with EEG data, we need to develop an understanding that EEG signals are extremely noisy by their nature; that patients are very heterogeneous; and that the ground truth (human raters and the human eye) can oftentimes not provide an unambiguous true label. Moreover, the question of coma outcome prognostication is a particularly challenging one, as it may suffer from self-fulfilling prophecies (2), or because patients' outcome may be determined by factors that cannot be measured by EEG, such as death following a second cardiac arrest, an infection, or non-medical circumstances such as personal directives or religious beliefs (for instance regarding withdrawal of life supporting treatment). Therefore, we must accept that there will always be cases where an algorithm cannot reach a decision with confidence, whether for technical (e.g., artifacts) or medical reasons. Algorithms should be offered the possibility to not provide a decision, instead of being forced to output a value. Additionally, algorithms in clinical settings should offer a measure of confidence in their single-patient prognostication, for instance by considering probabilities instead binary decisions. In fact, two studies have shown that the predicted “probability” output by the last networks layer correlates with actual probability (30) and with accuracy (29) of the prediction.

## 5. Conclusion

In summary, although promising results were obtained in research settings, deep learning has not yet revolutionized the field of clinical EEG evaluation, as it did in other domains, such as for example automatic translation. For future applications of AI in clinical practice, an interdisciplinary mindset must be cultivated and supported by clinical units, research institutions, and funding venues.

## Author contributions

All authors listed have made a substantial, direct, and intellectual contribution to the work and approved it for publication.
